# Multicopy integration of mini-Tn7 transposons into selected chromosomal sites of a Salmonella vaccine strain

**DOI:** 10.1111/1751-7915.12187

**Published:** 2014-12-09

**Authors:** Karen Roos, Esther Werner, Holger Loessner

**Affiliations:** Bacterial Vaccines and Immune Sera, Department of Veterinary Medicine, Paul Ehrlich InstituteLangen, 63225, Germany

## Abstract

Chromosomal integration of expression modules for transgenes is an important aspect for the development of novel *Salmonella* vectors. Mini-Tn7 transposons have been used for the insertion of one such module into the chromosomal site *attTn7*, present only once in most Gram-negative bacteria. However, integration of multiple mini-Tn7 copies might be suitable for expression of appropriate amounts of antigen or combination of different modules. Here we demonstrate that integration of a 9.6 kb mini-Tn7 harbouring the luciferase *luxCDABE* (*lux*) occurs at the natural *attTn7* site and simultaneously other locations of the *Salmonella* chromosome, which were engineered using λ-Red recombinase to contain one or two additional artificial *attTn7* sites (*a-attTn7*). Multicopy integration even at closely spaced *attTn7* sites was unexpected in light of the previously reported distance-dependent Tn7 target immunity. Integration of multiple copies of a mini-Tn7 containing a *gfp* cassette resulted in increasing green fluorescence of bacteria. Stable consecutive integration of two mini-Tn7 encoding *lacZ* and *lux* was achieved by initial transposition of *lacZ*-mini-Tn7, subsequent chromosomal insertion of *a-attTn7* and a second round of transposition with *lux*-mini-Tn7. Mini-Tn7 thus constitutes a versatile method for multicopy integration of expression cassettes into the chromosome of *Salmonella* and possibly other bacteria.

## Introduction

Engineering of whole bacterial genomes has advanced rapidly in the last decade (Carr and Church, 2009; Feher *et al*., 2012). However, combination of multiple functions and their orchestration remains challenging. Serial modification of bacterial genomes, such as chromosomal integration of large expression modules in a rapid, targeted and stable manner is difficult to achieve, especially in bacteria other than *Escherichia coli*. These difficulties also hamper the development of new recombinant live-attenuated bacterial vaccines such as *Salmonella enterica* ssp. (*Salmonella*) vector vaccines and the exploration of their potential as vector platform. Current strategies for rational design of these vaccines are based on a detailed understanding of bacteria–host interaction and the specific requirements for the induction of protective immune responses against a targeted pathogen (Galen *et al*., 2009; Hegazy and Hensel, 2012; Roland and Brenneman, 2013; Wang *et al*., 2013b; Galen and Curtiss, 2014). One approach for *Salmonella* vaccine strain development is the removal of genes in order to attenuate virulence, reduce metabolic burden and genetically stabilize or redirect intracellular trafficking of bacteria. Another strategy is based on programming of the bacteria for the delivery of a heterologous cargo, such as proteinaceous antigens or DNA vaccines. In this case, expression modules for one or several heterologous antigens in addition to other factors, such as lysis determinants, secretion system components, regulatory factors or adjuvant molecules, have to be stably introduced into the respective candidate strain. By combining these strategies, a vaccine strain was engineered to contain 13 gene deletions and two chromosomal integrated expression cassettes in addition to the plasmid-based DNA vaccine, yielding a self-destructing *Salmonella* vector system for efficient DNA vaccine delivery in mice (Kong *et al*., 2012). Although this provides an important proof of principle for *Salmonella* vector development, the recombinase-based suicide vector technology used to achieve these precise deletion/deletion–insertion mutations is cumbersome (Kang *et al*., 2002).

Alternatively, λ-Red recombineering has become a frequently used method for targeted gene deletions and chromosomal insertions of small DNA fragments (Sawitzke *et al*., 2007; Sharan *et al*., 2009). The polymerase chain reaction (PCR)-based version of this method (Datsenko and Wanner, 2000) allows deletion of chromosomal genes by transformation of an amplified DNA fragment, containing a selectable marker flanked by ∼ 50 bp extensions homologous to the chromosomal target sequence. In the presence of λ-Red recombinase, the specified chromosomal sequence is then replaced by the selectable marker gene in the recipient strain, which is subsequently removed. However, only relatively short additional heterologous DNA sequences can be chromosomally integrated this way due to a decrease of recombination efficiency for fragments exceeding ∼ 1500 bp (Datsenko and Wanner, 2000; Kuhlman and Cox, 2010). Chromosomal integration of larger fragments can still be achieved by λ-Red when long flanking homology regions are employed (Yu *et al*., 2011; Dharmasena *et al*., 2013; Sabri *et al*., 2013; Wang *et al*., 2013a), but such fragments cannot easily be generated by PCR. This obstacle can be overcome by a two-step procedure, in which λ-Red recombineering is combined with a second method. For example, λ-Red was used in the first step for the integration of a so-called landing pad into a selected chromosomal locus, which is a DNA fragment harbouring a selection marker flanked by I-SceI recognition sites and short sequences homologous to the integration cassette (Kuhlman and Cox, 2010). Subsequently, this integration cassette is transformed into bacteria, excised by I-SceI and recombined into the I-SceI-cleaved landing pad region, allowing the targeting of a 7 kb cassette into selected loci of the *E. coli* chromosome.

Mobile elements, such as transposon or phage-derived systems, integrate either randomly or site specifically into the bacterial genome and therefore do not allow deliberate targeting (Choi and Kim, 2009; Akhverdyan *et al*., 2011; Murphy, 2012; Loeschcke *et al*., 2013). Tn7-derived transposons, so-called mini-Tn7, have been frequently used for site-specific, single-copy integration of large DNA fragments into the chromosome of Gram-negative bacteria including *Salmonella* (Bao *et al*., 1991; Yan and Meyer, 1996; Choi *et al*., 2005; Loessner *et al*., 2007; Kvitko *et al*., 2013). In few bacteria, e.g. *Burkholderia* spp. or *Proteus mirabilis*, two or three natural sites have been found, which can be targeted by mini-Tn7 (Choi and Schweizer, 2006; Choi *et al*., 2006; 2014). In its natural form, Tn7 is a 14 kb transposable element encoding genes *tnsABCDE* of the transposition machinery and additional antibiotic resistance genes (Lichtenstein and Brenner, 1982; Peters and Craig, 2001). Recognition of its attachment site *attTn7*, located downstream of gene *glmS* in most bacteria including *Salmonella* spp., is mediated by the DNA-binding protein TnsD that recruits TnsC, an ATP-dependent DNA-binding protein, thereby linking target recognition with activation of transposase TnsAB (Bainton *et al*., 1993; Choi *et al*., 2014). Orientation-specific insertion of Tn7 is mediated by the asymmetric left and right transposon ends, Tn7L and Tn7R respectively. Both ends contain multiple TnsB binding sites. The presence of these sites in the chromosome as a consequence of transposon integration was described to confer so-called target immunity impeding further transposition events by a mechanism in which bound TnsB inactivates TnsC (Arciszewska *et al*., 1989; DeBoy and Craig, 1996; Stellwagen and Craig, 1997). This immunity effect was shown to be active at positions of the *E. coli* chromosome, which are located up to 190 kb apart from TnsB binding sites but integration into a site located 1900 kb away was not inhibited (DeBoy and Craig, 1996).

In this work, we have combined λ-Red recombineering with chromosomal integration of mini-Tn7 into the genome of the attenuated *S. enterica* serovar Typhimurium (*S.* Typhimurium) vaccine strain SL7207. We demonstrate that λ-Red-mediated integration of one or two artificial *attTn7* sites (*a-attTn7*) into chromosomal loci of choice allows subsequent integration of multiple mini-Tn7 copies simultaneously or consecutively into the bacterial genome. Chromosomal integration of one, two or three copies of a mini-Tn7 containing a *gfp* expression cassette allowed us to modulate GFP expression levels. Furthermore, consecutive integration of two different mini-Tn7 harbouring *lacZ* and *lux*, respectively, gave rise to a multifunctional strain.

## Results

**Fig 1:**
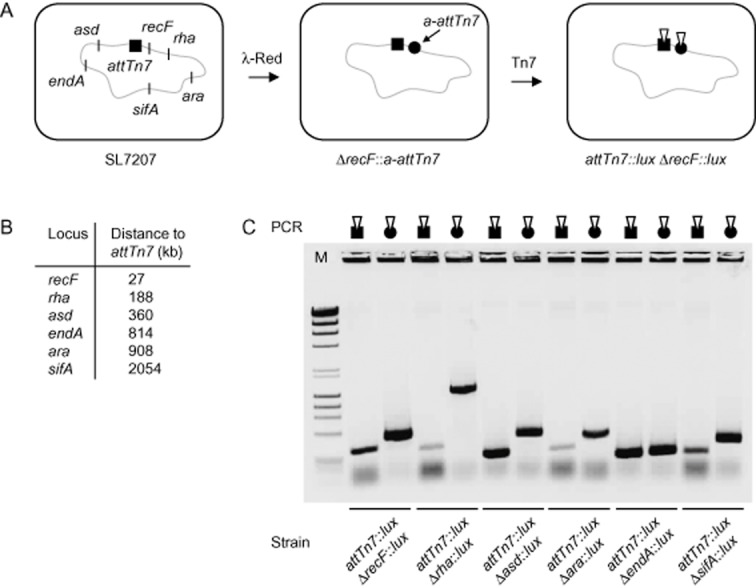
Integration of two *lux*-mini-Tn7 into *attTn*7 and in addition one *a-attTn*7 site inserted into selected chromosomal loci of *S*. Typhimurium.A. Strain SL7207 was modified in two steps for each of the indicated genes. λ-Red-mediated deletion and concurrent integration of *a-attTn*7 (black circle) is depicted for *recF*. Strains harbouring the natural *attTn*7 site (black square) and in addition *a-attTn*7 were used for transposition with *lux*-mini-Tn7 9.6 kb in size (white inversed triangle).B. Approximate distances between *attTn*7 and the newly introduced *a-attTn*7 sites in respective strains.C. Integration of *lux*-mini-Tn7 into *attTn*7 or *a-attTn*7 (indicated by combined symbols) was confirmed by the bioluminescent phenotype (data not shown) and by colony PCR with primers homologous to sequences of the right end of *lux*-mini-Tn7 (Tn7R) and the neighbouring genomic location (Supporting Information Table S1). The expected band sizes for *lux*-mini-Tn7 integration into *attTn*7 is 332 bp, for integrations into *a-attTn*7 sites in mutant strains are *recF* 586 bp, *rha* 1523 bp, *asd* 629 bp, *endA* 526 bp, *ara* 378 bp and *sifA* 543 bp respectively. Δ indicates a gene deletion and double colon indicates a chromosomal insertion. PCR data for representative clones are shown.

Mini-Tn7 transposons are frequently used for single-copy integration of DNA fragments into chromosomes of Gram-negative bacteria, with few exceptions (Choi *et al*., 2005; Crepin *et al*., 2012). However, consecutive integration of multiple mini-Tn7 copies into additional *attTn7* sites present in the *E. coli* chromosome was until now thought to be suppressed in a distance-dependent manner by Tn7 target immunity (Arciszewska *et al*., 1989; DeBoy and Craig, 1996; Stellwagen and Craig, 1997; Choi *et al*., 2014). Here we investigated the possibility of simultaneous integration of two mini-Tn7 copies into *attTn7* as well as an additional *a-attTn7* site inserted at genomic positions at varying distances apart from *attTn7* in the *S.* Typhimurium vaccine strain SL7207. The *a-attTn7* was derived from the *S.* Typhimurium strain SL1344 genomic sequence and contains 105 bp of the 3′ end of *glmS*, 140 bp intergenic region and 45 bp of the 3′ end of gene *SL1344_3827*. The *glmS* sequence harbours the entire TnsD binding site and the intergenic region *attTn7* downstream of *glmS* (Mitra *et al*., 2010). We successively replaced chromosomal genes *ara*, *asd*, *endA*, *recF*, *rha* and *sifA* of strain SL7207 with *a-attTn7* one at a time by λ-Red-mediated homologous recombination (Fig. 1A). The targeted loci were located at varying distances below or above 190 kb apart from *attTn7* (Fig. 1B). We then subjected the six different mutant strains carrying *attTn7* as well as one additional *a-attTn7* site to transposition with *lux*-mini-Tn7, which contains a constitutive *lux*-cassette and a kanamycin marker. Bioluminescent *Salmonella* colonies with chromosomal insertions of *lux*-mini-Tn7 were selected on LB plates containing streptomycin and kanamycin. Colony PCR of all mutant strains revealed that integration of *lux*-mini-Tn7 occurred into both integration sites (Fig. 1C). This was even the case for strains carrying *a-attTn7* in the *recF* and the *rha* loci, which are less than 190 kb away from *attTn7*. Therefore, we did not observe immunity-related inhibition of *lux*-mini-Tn7 integration into either one of the two transposon integration sites present in the genome of all tested mutant strains.

**Fig 2:**
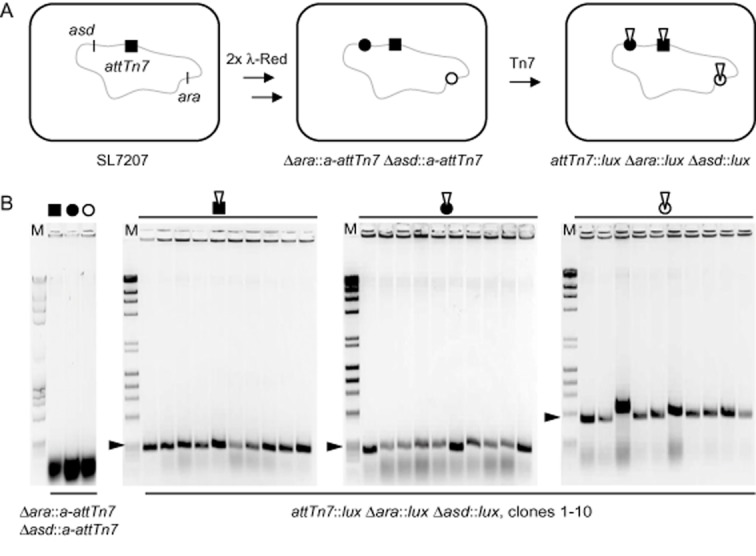
Consistency of simultaneous transposition of *lux*-mini-Tn7 into three loci of the *S*. Typhimurium chromosome.A. A SL7207 derivative with the natural *attTn*7 site (black square) and two additional *a-attTn*7 sites (black and white circles) was generated by two rounds of λ-Red-mediated recombination linked with chromosomal integration of the *a-attTn*7 sequence each time.B. Strain SL7207Δ*ara*::*a-attTn*7Δ*asd*::*a-attTn*7 harbouring three sites for Tn7 transposition was used for transposition with *lux*-mini-Tn7 (white inversed triangle). *lux*-mini-Tn7 integration into the three sites was confirmed by colony PCR for 10 bioluminescent clones selected on LB plates containing streptomycin and kanamycin. The strain harbouring three empty Tn7 transposition sites was used as control template. The band size for *lux*-mini-Tn7 integration into *attTn*7 is 332 bp and for integrations into *a-attTn*7 are *ara* 378 bp and *asd* 629 bp, band positions are indicated by black arrow heads.

To investigate simultaneous integration of *lux*-mini-Tn7 into additional chromosomal locations, *a-attTn7* sites were integrated into *ara* and *asd* loci by two subsequent rounds of λ-Red-mediated gene replacement (Fig. 2A). The intermediate strain harbouring three Tn7 integration sites was then again subjected to transposition with *lux*-mini-Tn7 (Fig. 2A). In this strain the three Tn7 integration sites were spaced more than 190 kb apart from each other. PCR analysis of colonies obtained from selective plates revealed that mini-Tn7 integration occurred consistently into all three transposon integration sites in all tested colonies (Fig. 2B). Such clones could also be identified due to the bioluminescent phenotype alone by plating a dilution series of the triple mating culture on LB plates containing only streptomycin. The mean frequency of identified positive colonies from three experimental repetitions was (4.5 ± 1.7) × 10^−5^, indicating that high efficiency of multicopy integration can even be achieved with a mini-Tn7 devoid of an antibiotic resistance marker if phenotypical identification of colonies is possible.

**Fig 3:**
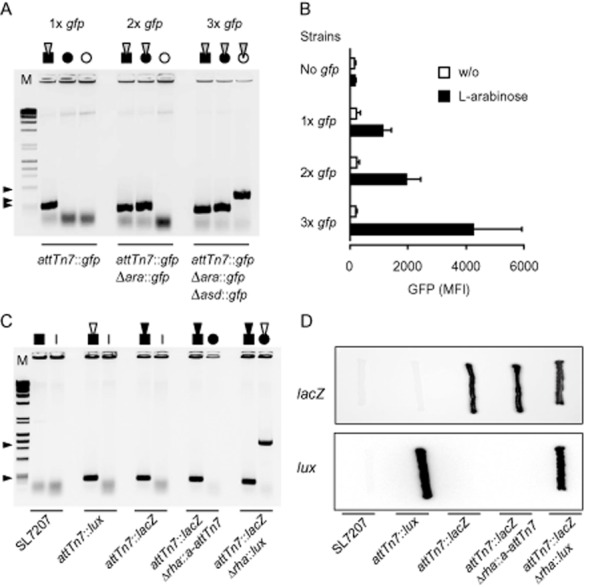
Modulation of GFP synthesis by chromosomal integration of either one, two or three copies of *gfp*-mini-Tn7 and consecutive chromosomal integration of two different mini-Tn7 encoding *lacZ* and *lux* into *S*. Typhimurium strain SL7207.A. Strain SL7207 with the natural *attTn*7 site (black square) or derivatives with one or two additional *a-attTn*7 sites integrated into *ara* and *asd* loci (black and white circles) were used for transposition with *gfp*-mini-Tn7 harbouring the L-arabinose inducible *gfp* expression cassette (grey inversed triangle). *gfp*-mini-Tn7 integration into each site was verified by colony PCR and expected bands were obtained at 332 bp for integration into *attTn*7, 378 bp for integration into *a-attTn*7 at the *ara* locus and 629 bp for integration into *a-attTn*7 at the *asd* locus. Bands are indicated by black arrow heads.B. Strains SL7207 and derivatives with one, two or three chromosomal copies of the *gfp* cassette were grown in the absence or presence of L-arabinose prior to flow cytometric analysis. Depicted mean fluorescence intensities (MFI) represent three experimental repetitions and error bars indicate standard deviation.C. Strain SL7207 was used for transposition with *lacZ*-mini-Tn7 or *lux*-mini-Tn7 (black and white inversed triangles). Recipient strains carry either *lacZ* or *lux* within *attTn*7 (black square) and are designated *attTn*7::*lacZ* and *attTn*7::*lux* respectively. Subsequently, the *rha* operon of strain *attTn*7::*lacZ* was replaced by *a-attTn*7 (white square) giving rise to strain *attTn*7::*lacZ*Δ*rha*::*a-attTn*7. This strain was employed for another transposition round with *lux*-mini-Tn7 yielding strain *attTn*7::*lacZ*Δ*rha*::*lux*. Colony PCR verified transposon integrations into the chromosome of each strain. Band sizes for integration into *attTn*7 are 332 bp and into *a-attTn*7 at the *rha* locus 1523 bp.D. *lacZ* expression of bacteria was observed on X-Gal plates by blue dye formation and *lux* expression by detection of bioluminescence from the same plate.

To determine if chromosomal integration of multiple copies of a *gfp*-mini-Tn7 allows a stepwise, copy number-dependent increase in the expression level of a transgene, we integrated one, two or three copies of a GFP expression cassette into SL7207. Towards this, either one or two additional *a-attTn7* sites were initially recombined into the *ara* and *asd* loci, and then a *gfp*-mini-Tn7 was used for transposition of respective strains. Colonies containing insertions within the present Tn7 integration sites were identified by colony PCR (Fig. 3A). These clones were then grown in liquid medium and green fluorescence of cells was analysed by flow cytometry upon induction of GFP synthesis by addition of L-arabinose. The level of green fluorescence intensity measured correlated with the copy number of chromosomally integrated *gfp*-mini-Tn7 at selected loci, while cultures grown in the absence of L-arabinose displayed only low GFP fluorescence (Fig. 3B and Supporting Information Fig. S1).

In addition to the integration of multiple copies of the same mini-Tn7, we also investigated the consecutive integration of two different mini-Tn7 carrying *lacZ* and *lux* into strain SL7207. In a first round of transposition, either *lacZ*-mini-Tn7 or *lux*-mini-Tn7 was integrated into the native Tn7 recognition site *attTn7*. Thereafter, the strain harbouring *lacZ*-mini-Tn7 was subjected to λ-Red-mediated integration of *a-attTn7* into the *rha* locus. Transposition of *lux*-mini-Tn7 into this strain gave rise to bacteria harbouring both mini-Tn7 encoding *lacZ* and *lux* (Fig. 3C). Phenotypical analysis of these strains confirmed the expression of *lacZ*, *lux* or both transgenes (Fig. 3D). To assess the stability of *lacZ* and *lux* expression cassettes we evaluated colonization and foreign gene expression of the recombinant *S.* Typhimurium strains in BALB/c mice. Upon oral administration, tissue colonization of bioluminescent SL7207 bacteria peaks around day 7 after oral administration (Burns-Guydish *et al*., 2005). Groups of five mice were orally inoculated with the original strain SL7207 and derivatives *attTn7*::*lux* and *attTn7*::*lacZ*Δ*rha*::*lux*. The *in vivo* colonization course of the two bioluminescent strains was followed by non-invasive *in vivo* imaging for 9 days (Supporting Information Fig. S2A). Mice were then sacrificed, and Peyer's patches, mesenteric lymph nodes and spleens were harvested for determination of colony-forming units (Supporting Information Fig. S2B). We found that all tested strains readily colonized the analysed organs, even though the strains expressing additional transgenes yielded lower numbers, indicating an attenuating effect. However, all engineered bacteria retained *lacZ* and *lux* expression as confirmed by phenotypical examination of bacterial colonies recovered from organs (Supporting Information Fig. S2B), illustrating that consecutive transposition of different mini-Tn7 is a feasible approach for stable chromosomal integration of various heterologous expression cassettes into *Salmonella* and should also be considered for other Gram-negative bacteria.

## Discussion

Recombinant *Salmonella* vector vaccines stimulate humoral and cell-mediated immune responses in mucosal and systemic compartments to self-antigens as well as to heterologous antigens derived from infectious agents or malignant tissues (Galen *et al*., 2009; Curtiss *et al*., 2010; Paterson *et al*., 2010; Hegazy and Hensel, 2012; Roland and Brenneman, 2013; Toussaint *et al*., 2013; Wang *et al*., 2013b). The potency of such a vaccine depends on its ability to induce high-level production of the respective antigens once bacteria have reached the immune inductive sites. Therefore, the introduction of appropriately tailored antigen expression cassettes is an important step during construction of a recombinant *Salmonella* vector vaccine. The use of a self-replicating plasmid is one option for the introduction of such cassettes. Such plasmids are designed to assure stable retention by the recipient bacteria and to maintain colonization of immune inductive tissues (Bauer *et al*., 2005; Galen *et al*., 2010; Xin *et al*., 2012). However, this situation is difficult to achieve with multicopy plasmids harbouring expression cassettes for large gene clusters, but these limitations can be overcome by integrating such cassettes into the bacterial chromosome (Husseiny and Hensel, 2008; Yu *et al*., 2011; Dharmasena *et al*., 2013).

A variety of transposons have been used as gene delivery tools for bacteria and eukaryotic cells (Choi and Kim, 2009; Ivics and Izsvak, 2011). Tn7 is the hallmark transposon for site-specific single-copy integration into the chromosome of Gram-negative bacteria (Peters and Craig, 2001). Here we investigated the possibility of integrating multiple copies of a mini-Tn7 into the chromosome of the *S.* Typhimurium strain SL7207 containing several *attTn7* sites. Towards this, we used λ-Red recombineering for the insertion of initially one *a-attTn7* site into selected positions of the *Salmonella* chromosome located at varying distances from *attTn7*. Using a 9.6 kb mini-Tn7 with a constitutive luciferase expression cassette, we obtained for each position transposon insertions at both sites *attTn7* and *a-attTn7*. Surprisingly, this was independent from the distance between the sites, which was as small as 27 kb in the case of the *recF* locus and as large as 2054 kb in the case of the *sifA* locus. Integration of three *lux*-mini-Tn7 copies into *attTn7* and two additional *a-attTn7* sites located in the *asd* and *ara* loci occurred with the same consistency. To our knowledge, this is the first report of integration of multiple mini-Tn7 copies into deliberately selected loci of the bacterial chromosome down to a distance below 190 kb between integration sites, which was previously considered inefficient due to Tn7 target immunity (DeBoy and Craig, 1996). Chromosomal integration of multiple mini-Tn7 copies has been reported to occur naturally in *Burkholderia* ssp. and *P. mirabilis*, which carry two or three natural integration sites in their bacterial chromosomes (Choi and Schweizer, 2006; Choi *et al*., 2006; 2008). For example in *Burkholderia pseudomallei* strain 1026b three natural *attTn7* sites are present, which are located downstream of three *glmS* genes (Choi *et al*., 2008). Mini-Tn7 integration occurs in > 65% of events as single-copy integrations downstream of gene *glmS2*. The other two *attTn7* sites downstream of genes *glmS1* and *glmS3* were targeted by single-copy integration of mini-Tn7 at much lower rates. Double insertions of a mini-Tn7 in one cell occur at a rate of 10–20%, but triple insertions at once were only rarely observed (Choi *et al*., 2008). The reason of the low double and triple mini-Tn7 insertion frequency in this strain is unknown. Tn7 target immunity would not be expected to be the obvious reason as *attTn7* sites on chromosome 1 are 1,1 Mb apart from each other, and one *attTn7* site is located on chromosome 2. In *P. mirabilis* strain HI4320, Choi and Schweizer (2006) always observed simultaneous mini-Tn7 insertion into two sites, the *attTn7* site downstream of *glmS* and one site located in *carA*, when bacteria were grown in rich medium. These sites are spaced 724 kb and would also not expected to be affected by Tn7 target immunity. However, in our work with *Salmonella* we observed a frequency of 100% of simultaneous mini-Tn7 insertions at two or three *attTn7* sites, even in the case of closely spaced *attTn7* sites 27 or 188 kb apart.

Designing *Salmonella* vector vaccines for the immunization against heterologous pathogens has so far required a number of precise chromosomal gene deletions and module integrations in parallel. The combination of λ-Red-mediated gene deletion with mini-Tn7 integration reported here has the potential to considerably facilitate this process. As target loci for mini-Tn7 integration, we specifically selected genes, which have been previously removed from *Salmonella* vaccine strains as such sites should be suited for the stable integration of heterologous expression cassettes. Δ*asd* strains were used as hosts for plasmids stabilized by the complementing essential *asd* gene, referred to as balanced lethal systems (Nakayama *et al*., 1988). In addition, the Δ*asd* mutation is present in strains undergoing a so-called delayed type of bacterial lysis (Kong *et al*., 2008). Δ*ara* and Δ*rha* mutations have been introduced into vaccine strains in order to prevent degradation of L-arabinose or L-rhamnose when such sugars are used as inductors of gene expression (Kong *et al*., 2008). Δ*recE* strains were shown to display a reduced capacity for recombination and therefore are suited for stable maintenance of dual-plasmid systems in vaccine strains (Zhang *et al*., 2011; Xin *et al*., 2012). Δ*sifA* strains were employed for the cytosolic delivery of heterologous antigens or DNA vaccines due to their ability to escape the phagosomal compartment inside host cells (Brumell *et al*., 2002). When we used a *gfp*-mini-Tn7, integration of one, two or three copies into chromosomal loci *attTn7*, Δ*ara*::*a-attTn7* and Δ*asd*::*a-attTn7*, respectively, correlated with a stepwise increase of GFP fluorescence demonstrating a stepwise modulation of the expression level by targeted multicopy integration of a mini-Tn7.

Live bacterial vaccine or vector construction often necessitates chromosomal integration of expression cassettes for different transgenes (Galen *et al*., 2009; Curtiss *et al*., 2010; Hegazy and Hensel, 2012; Wang *et al*., 2013b). Therefore, we investigated the consecutive introduction of two different mini-Tn7 harbouring reporter genes *lacZ* or *lux* into a strain carrying *attTn7* and *a-attTn7* respectively. The recombinant bacteria containing both modules were readily obtained by initial transposition of *lacZ*-mini-Tn7, followed by λ-Red-mediated replacement of the *rha* locus with *a-attTn7* and subsequent transposition of the *lux*-mini-Tn7. Importantly, *lacZ* and *lux* expression was stably retained during colonization of mouse tissues upon oral administration. However, instability of such modules might potentially be caused by endogenous recombinases acting on homologous sequences such as attTn7/a-attTn7, but even more so between multiple copies of the same mini-Tn7. This problem might be circumvented by the removal of host recombinases, reduction of sequence homologies and appropriate chromosomal positioning of insertion sequences (Zhang *et al*., 2011). Bioluminescence *in vivo* imaging and subsequent recovery and quantification of bacteria from mice indicated that the strains containing mini-Tn7 insertions colonized tissues to a somewhat lower extent possibly due to an attenuating effect of constitutive expression of *lacZ* and *lux*. However, combination of our approach with strategies for the inducible expression of heterologous factors may be able to resolve such effects (Hohmann *et al*., 1995).

In conclusion, we consider λ-Red recombinase-mediated positioning of additional Tn7 attachment sites in the bacterial genome followed by mini-Tn7 transposition a versatile method for the construction of multifunctional *Salmonella* vaccines and vectors. Further work should be devoted to answer questions such as (i) how many mini-Tn7 copies can be reliably integrated and (ii) will consecutive insertions in close proximity be compromised by Tn7 target immunity. The ability of researchers to construct multifunctional vector strains may help to overcome known limitations of present recombinant *Salmonella* vaccines and eventually allow the development of a new generation of efficacious and safe *Salmonella* vaccines for clinics (Galen *et al*., 2009; Wang *et al*., 2013b). Moreover, such novel *Salmonella* vector systems also constitute promising delivery systems for other medical interventions, such as tumour therapy (Forbes, 2010; Leschner and Weiss, 2010).

## Experimental procedures

### Bacterial strains and growth conditions

*Salmonella enterica* serovar Typhimurium strain SL7207 (Δ*aroA*, Δ*hisG*) was originally obtained from Bruce Stocker (Hoiseth and Stocker, 1981) and kindly provided by Siegfried Weiss (Helmholtz Center for Infection Research, Braunschweig). Chromosomal modification of this strain was mediated by λ-Red recombinase or transposition of a mini-Tn7 (see below). Strain derivatives are described in the Results section. *Escherichia coli* strains Top10 and Pir2 (Life Technologies) were used as hosts for cloning, and *E. coli* strain SM10*λpir* (Simon *et al*., 1983) was used for mobilization of mini-Tn7 and the Tn7 helper plasmid. Bacteria were grown on LB agar plates or in LB medium supplemented with 100 μg ml^−1^ ampicillin, 30 μg ml^−1^ kanamycin, 30 μg ml^−1^ streptomycin, 2 mg ml^−1^ L-arabinose, 50 μg ml^−1^ diaminopimelic acid or 30 μg ml^−1^ 5-Bromo-4-chloro-3-indolyl-β-D-galactopyranoside (X-Gal), where appropriate. LB medium base and supplements were purchased from Carl Roth.

### Plasmid constructions

A 300 bp sequence harbouring the *attTn7* integration site (*a-attTn7*) was derived from the genomic sequence of strain *S.* Typhimurium SL1344 obtained from NCBI GenBank (accession number FQ312003, position 4090151–4090450 bp). This sequence including flanking EcoRI restriction sites was synthesized and sequenced (Geneart, Life Technologies). To apply λ-Red recombination for chromosomal integration of *a-attTn7*, the template plasmid pKD4 (Datsenko and Wanner, 2000) was modified. The synthetic DNA was cleaved with EcoRI and inserted into the NdeI site of plasmid pKD4 after ends were made compatible with Klenow enzyme. The plasmid containing the insert in the same orientation as the kanamycin resistance gene was named pKR31a. This plasmid was subsequently used as template for the generation of PCR products with homology ends for λ-Red recombination. Primer pairs for the replacement of *ara*, *asd*, *endA*, *recF*, *rha* and *sifA* are listed in Supporting Information Table S1. Plasmids pKD46 (Datsenko and Wanner, 2000) and pCP20 (Cherepanov and Wackernagel, 1995) were used for the expression of λ-Red (*exo*, *bet* and *gam* genes) and FLP recombinase respectively. Expression cassettes for the bioluminescence operon *luxCDABE* (*lux*) derived from *Photorhabdus luminescence*, the green fluorescence protein mutant 2 variant (*gfp*) (Cormack *et al*., 1996) and β-galactosidase of *E. coli* (*lacZ*) were cloned into a derivative of the mini-Tn7 pUX-BF5 (Bao *et al*., 1991). The constitutive *lux* expression cassette was originally subcloned from plasmid pLite201 (Voisey and Marincs, 1998) into plasmid pHL300 (Loessner *et al*., 2009). This plasmid was cut with BspLUII, blunt ended and again digested with BsrGI. The 1307 bp partial *lux* fragment was subsequently inserted into the mini-Tn7 transposon plasmid pHL289 (Loessner *et al*., 2007). For this, plasmid pHL289 was opened with NheI, blunt ended and thereafter cut with BsrGI. The product was designated pHL305 (*lux*-mini-Tn7). *gfp* linked to the L-arabinose inducible promoter P_BAD_ (Loessner *et al*., 2008) was cloned into a derivative of pUX-BF5 giving rise to plasmid pKR49 (*gfp*-mini-Tn7). *lacZ* was amplified from plasmid pCMVβ with *lacZ* forward and reverse primers (Supporting Information Table S1). The PCR fragment was digested with XbaI and HindIII and inserted into the same restriction sites of plasmid pHL222 (Loessner *et al*., 2006) giving rise to plasmid pHL325. *lacZ* in conjunction with the constitutive β-lactamase promoter of *E. coli* was obtained from this plasmid by cleavage with SmaI and HindIII and inserted into the mini-Tn7 plasmid pKR50 opened with SalI and MluI after ends were blunted. The construct was designated pKR61 (*lacZ*-mini-Tn7). Plasmid pUX-BF13 containing Tn7 transposon genes *tnsABCDE* was used as helper plasmid for transposition of mini-Tn7 (Bao *et al*., 1991). Primers used for confirmation of chromosomal integrations of mini-Tn7 are listed in Supporting Information Table S1.

### λ-Red recombinase-mediated gene replacement

λ-Red recombinase-mediated gene replacement was carried out as previously described (Datsenko and Wanner, 2000). Briefly, PCR products harbouring ∼ 40 bp end sequences homologous to the respective *S.* Typhimurium target genes, a kanamycin resistance marker flanked by FLP recombinase recognition sites, and *a-attTn7*, so-called Red knock out (ko) fragments, were amplified with pKR31a as template and so-called Red ko primer (Supporting Information Table S1). Strain SL7207 harbouring plasmid pKD46 was grown in liquid LB medium supplemented with ampicillin at 30°C and 200 r.p.m. agitation up to an optical density at 600 nm (OD600) of ∼ 0.4. At this time point λ-Red recombinase expression was induced by addition of L-arabinose, and 1 h later electro-competent cells were prepared by washing cells three times in ice-cold distilled water. An amount of approximately 200 ng of the Red ko fragment was electroporated into cells with the Gen Pulser Xcell System (Bio-Rad) at 2.5 kV, 400 Ohm and 25 μF using a 0.2 cm cuvette. SL7207 mutant clones were selected on media plates containing kanamycin and streptomycin at 37°C. The kanamycin resistance gene was then removed by action of FLP recombinase as previously described (Datsenko and Wanner, 2000).

### Transposition of mini-Tn7 into *S*. Typhimurium

Transposon-mediated site-specific integration into the chromosome of strain SL7207 was carried out according to the method of Bao *et al*. (1991). Briefly, triple mating cultures consisting of the helper strain *E. coli* SM10*λpir* harbouring pUX-BF13, *E. coli* SM10*λpir* harbouring a mini-Tn7 plasmid and the *S.* Typhimurium strain SL7207 were incubated on non-selective medium plates at 30°C for 24 h. Thereafter, bacteria were recovered and plated on medium plates containing streptomycin, kanamycin or chloramphenicol respectively. Site-specific chromosomal integration was verified by colony PCR (see Supporting Information). Of note, plasmid pUX-BF13 mediates synthesis of TnsABCDE proteins. TnsABC + D proteins are sufficient for target-specific chromosomal insertion of mini-Tn7 into *attTn7* (Waddell and Craig, 1988). In contrast TnsABC + E mediated mini-Tn7 insertion occurs at non-*attTn7* sites, but only on conjugal plasmids (Wolkow *et al*., 1996). Therefore, we do not expect interference of TnsE expression with the chromosomal mini-Tn7 insertions we observe in this work.

### Flow cytometry

Bacteria were grown at 37°C and 200 r.p.m. agitation up to OD600 of ∼ 0.6. Then *gfp* expression was induced by addition of L-arabinose and 1 h later bacteria were pelleted, suspended in phosphate-buffered saline and subsequently analysed on a BD Accuri C6 flow cytometer (Becton Dickinson) as previously described (Loessner *et al*., 2006). An appropriate scatter gate was used to distinguish bacteria from other particles. Data were acquired and analysed with BD CSampler software (Becton Dickinson).

### Animal work and ethics statement

Experimental procedures for the work with mice are provided as Supporting Information. All animal experiments were in compliance with the German Animal Welfare Act and approved by the competent authority (Regierungspraesidium Darmstadt).

## Supporting Information

**Fig. S1.** Modulation of GFP synthesis in *S.* Typhimurium strain SL7207 by chromosomal integration of either one, two or three copies of *gfp*-mini-Tn7 (Fig. 3A,B). Strains SL7207 and derivatives with one, two or three chromosomal copies of the *gfp* cassette were induced with L-arabinose and subsequently analysed by flow cytometry. The histogram shows data of one representative experiment. **Fig. S2.** Consecutive chromosomal integration of two different mini-Tn7 encoding *lacZ* and *lux* into *S.* Typhimurium and stable maintenance of both modules by bacteria during colonization of mice. A. 10^9^ bacteria of strains SL7207, *attTn7::lux* and *attTn7:lacZΔrha::lux* (Fig. 3C,D) were orally administered to BALB/c mice (*n* = 5 each group). The colonization course of bioluminescent strains harbouring *lux* was followed by non-invasive *in vivo* imaging. Bioluminescence intensities of abdominal regions of mice are expressed as means of radiance and error bars indicate standards deviations. B. At day 9 post-infection (p.i.) Peyer's patches (PP), mesenteric lymph nodes (MLN) and spleens were harvested (*n* = 5 each group), and bacterial content of organs was determined by plating dilution series of tissue homogenates on X-Gal plates. Data are presented as means of cfu with error bars indicating standard deviations. Stability of *lacZ* and *lux* cassettes was judged based on the blue and bioluminescent colony phenotype. **Table S1.** Primers used in this study. **Appendix S1.** Experimental procedures.
