# Supplementary material for: Multicopy integration of mini-Tn7 transposons into selected chromosomal sites of a Salmonella vaccine strain
Source: Microb Biotechnol. 2014 Dec 9;8(1):177–87. doi: 10.1111/1751-7915.12187 (PMC4321384; doi:10.1111/1751-7915.12187)
Supplement: Supplementary file 1 — Fig. S1. Modulation of GFP synthesis in S. Typhimurium strain SL7207 by chromosomal integration of either one, two or three copies of gfp-mini-Tn7 (Fig. 3A,B). Strains SL7207 and derivatives with one, two or three chromosomal copies of the gfp cassette were induced with L-arabinose and subsequently analysed by flow cytometry. The histogram shows data of one representative experiment. Fig. S2. Consecutive chromosomal integration of two different mini-Tn7 encoding lacZ and lux into S. Typhimurium and stable maintenance of both modules by bacteria during colonization of mice. A. 109 bacteria of strains SL7207, attTn7::lux and attTn7:lacZΔrha::lux (Fig. 3C,D) were orally administered to BALB/c mice (n = 5 each group). The colonization course of bioluminescent strains harbouring lux was followed by non-invasive in vivo imaging. Bioluminescence intensities of abdominal regions of mice are expressed as means of radiance and error bars indicate standards deviations. B. At day 9 post-infection (p.i.) Peyer's patches (PP), mesenteric lymph nodes (MLN) and spleens were harvested (n = 5 each group), and bacterial content of organs was determined by plating dilution series of tissue homogenates on X-Gal plates. Data are presented as means of cfu with error bars indicating standard deviations. Stability of lacZ and lux cassettes was judged based on the blue and bioluminescent colony phenotype. Table S1. Primers used in this study. Appendix S1. Experimental procedures. [file mbt20008-0177-sd1.zip › mbt212187-supp-0001-tableS1.docx]

**Supplementary information Roos et al.**

**Experimental procedures**

Polymerase chain reaction (PCR)

For PCR the OneTaq 2x Master Mix (New England Biolabs) was used. Approximately 2 kb Red ko fragments were amplified in a 20 µl reaction mixture containing 1x master mix, 0.2 µM of each Red ko forward and reverse primer and 30 ng of the template plasmid pKR31a. The PCR reaction was started with an initial denaturation step at 94 °C for 2 min and continued with 30 cycles of denaturation at 94 °C for 20 s, annealing at 55 °C for 20 s, and elongation at 68 °C for 120 s. The reaction was concluded with a final elongation step at 68 °C for 5 min. Reaction mixtures were purified with the QIAquick PCR Purification Kit (Qiagen). Chromosomal integrations mediated by -Red recombination or Tn7 transposition were confirmed by colony PCR. Here, individual colonies were picked and directly suspended into the 20 µl PCR reaction mix containing 1x master mix and 0.2 µM of each so called check primer (Table S1). The PCR was started with an initial denaturation step at 94 °C for 5 min and continued with 35 cycles of denaturation at 94 °C for 20 s, annealing at 60 °C for 20 s and elongation at 68 °C for 60 s. The reaction was again concluded with a final elongation step at 68 °C for 5 min. Afterwards the whole reaction volume was directly loaded on TAE agarose gels for electrophoresis together with 5 µl of the  Hind III / phiX Hae III DNA Marker (Carl Roth).Table S1. Primers used in this study

| Target | 5’ - 3’ sequence |
| --- | --- |
| ***Red ko primer*** |  |
| *ara ko fw* | CAATTGCAATTGGCCTCGATTTTGGCAGTGATTCAGTGCGCGCTCTGTGTAGGCTGGAGCTGCTTC |
| *ara ko rev* | GTGTTTGCGTAGATAGTGTTTATCCAGCAGGGATTGCTGCATGTCGATGTAACGCACTGAGAAGC |
| *asd ko fw* | ATGGTGAAGGATGCGCCACAGGATACTGGCGCGCATACACAGTGTGTAGGCTGGAGCTGCTTC |
| *asd ko rev* | CTACGCCAACTGGCGCAGCATTCGACGCAGCGGCTCGGCGGCGATGTAACGCACTGAGAAGC |
| *endA ko fw* | CGTTGCGGGCTCGTTTTGCTATGGAGTGTGCAATGTACCGTGTGTAGGCTGGAGCTGCTTC |
| *endA ko rev* | CACATAGGGATTATGATTACCCTGGACCTTCGCGATACGCGATGTAACGCACTGAGAAGC |
| *recF ko fw* | TTAATCCGTTATTTTACCCTTTTCCACGGTAAACATCTTCTGTGTAGGCTGGAGCTGCTTC |
| *recF ko rev* | TGTCACTGACGCGCCTTTTAATCAAAGACTTCCGCAACATGATGTAACGCACTGAGAAGC |
| *rha* *ko fw* | TCAGTACAGCGCTACGGCGCTGGCCAGCGGTGTCACACCAAAACGTTTGCCGAGTGCGACTGTGTAGGCTGGAGCTGCTTC |
| *rha* *ko rev* | ATGACTTTTCGCCATTGTGTCGCGGTTGATCTCGGCGCATCCAGCGGGCGCGTGATGCTGGATGTAACGCACTGAGAAGC |
| *sifA ko fw* | TTATAAAAAACAACATAAACAGCCGCTTTGTTGTTCTGAGTGTGTAGGCTGGAGCTGCTTC |
| *sifA ko rev* | ATGCCGATTACTATAGGGAATGGTTTTTTAAAAAGTGAAAGATGTAACGCACTGAGAAGC |
| ***Check primer for mini-Tn7 integration*** |  |
| Tn7R check | CACAGCATAACTGGACTGATTTC |
| *glmS check* | CGGTCAGTTGTACGTCTTC |
| *ara check* | CCGTGTTTGCGTAGATAGTG |
| *asd check* | TATCTGCGTCGTCCTACCTTCAG |
| *endA check* | CGTACCGGACGTCAACAATTC |
| *recF check* | CAGCGTACAGATTGAGGATG |
| *rha* *check* | CGTTCGCGTTACTGGATTATCG |
| *sifA check* | AGCGACGCTATCACAGACAG |
| ***Cloning primer*** |  |
| *lacZ fw* | gctctagatttaagaaggagatatacatatgtcgtttactttgaccaa |
| *lacZ rev* | gattacccaagctttattatttttgacaccagacc |

Oral immunization and non-invasive bioluminescence imaging of mice

Female BALB/c mice 6 weeks of age were purchased (Janvier) and acclimatized for 1-2 weeks. Bacteria were grown in LB medium at 37 °C with 100 rpm agitation up to an OD600 of 1, pelleted, resuspended in phosphate buffered saline, and adjusted to 1010 colony forming units (cfu) ml-1. After removal of food and drinking water for 1 h, 50 µl of 0.6 M sodium bicarbonate was administered orally to each mouse. Thereafter, mice were gavaged with 100 µl of the bacterial solution, and food and water were replaced. The colonization course of luminescent S. typhimurium was followed by non-invasive bioluminescence imaging using the IVIS-Spectrum system (PerkinElmer). Towards this, mice were anaesthetized with isoflurane using the XGI-8 gas anesthesia system (PerkinElmer). The software Living Image 4.1 (PerkinElmer) was used for image analysis and quantification of bioluminescence intensities of abdominal regions of mice.

Recovery of bacteria from mouse tissues

At day nine post-infection, mice were sacrificed and spleens, mesenteric lymph nodes, and Peyer’s patches were transferred individually into 3 ml of sterile ice-cold in phosphate buffered saline containing 0.1 % (v/v) Triton X-100. Tissues were disrupted using an Ultra Turrax homogenizer (IKA), and the numbers of recovered bacteria were determined by plating of serially diluted homogenates on X-Gal medium plates and subsequent counting of colonies. Observation of blue and bioluminescent colonies confirmed expression of lacZ and lux.
