# Supplementary figures and images for: Multicopy integration of mini-Tn7 transposons into selected chromosomal sites of a Salmonella vaccine strain
Source: Microb Biotechnol. 2014 Dec 9;8(1):177–87. doi: 10.1111/1751-7915.12187 (PMC4321384; doi:10.1111/1751-7915.12187)

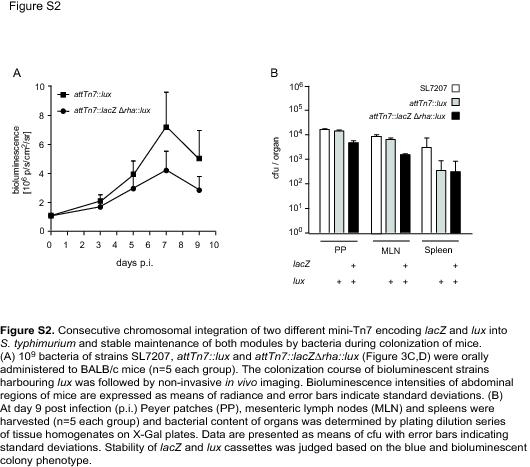

Supplement: Supplementary file 1 — Fig. S1. Modulation of GFP synthesis in S. Typhimurium strain SL7207 by chromosomal integration of either one, two or three copies of gfp-mini-Tn7 (Fig. 3A,B). Strains SL7207 and derivatives with one, two or three chromosomal copies of the gfp cassette were induced with L-arabinose and subsequently analysed by flow cytometry. The histogram shows data of one representative experiment. Fig. S2. Consecutive chromosomal integration of two different mini-Tn7 encoding lacZ and lux into S. Typhimurium and stable maintenance of both modules by bacteria during colonization of mice. A. 109 bacteria of strains SL7207, attTn7::lux and attTn7:lacZΔrha::lux (Fig. 3C,D) were orally administered to BALB/c mice (n = 5 each group). The colonization course of bioluminescent strains harbouring lux was followed by non-invasive in vivo imaging. Bioluminescence intensities of abdominal regions of mice are expressed as means of radiance and error bars indicate standards deviations. B. At day 9 post-infection (p.i.) Peyer's patches (PP), mesenteric lymph nodes (MLN) and spleens were harvested (n = 5 each group), and bacterial content of organs was determined by plating dilution series of tissue homogenates on X-Gal plates. Data are presented as means of cfu with error bars indicating standard deviations. Stability of lacZ and lux cassettes was judged based on the blue and bioluminescent colony phenotype. Table S1. Primers used in this study. Appendix S1. Experimental procedures. [file mbt20008-0177-sd1.zip › Figure S2.jpg]
